# Supplementary material for: Absence of central tolerance in Aire-deficient mice synergizes with immune-checkpoint inhibition to enhance antitumor responses
Source: Commun Biol. 2020 Jul 8;3:355. doi: 10.1038/s42003-020-1083-1 (PMC7343867; doi:10.1038/s42003-020-1083-1)
Supplement: Supplementary file 1 — Supplementary Information [file 42003_2020_1083_MOESM1_ESM.pdf]

Supplementary Figures

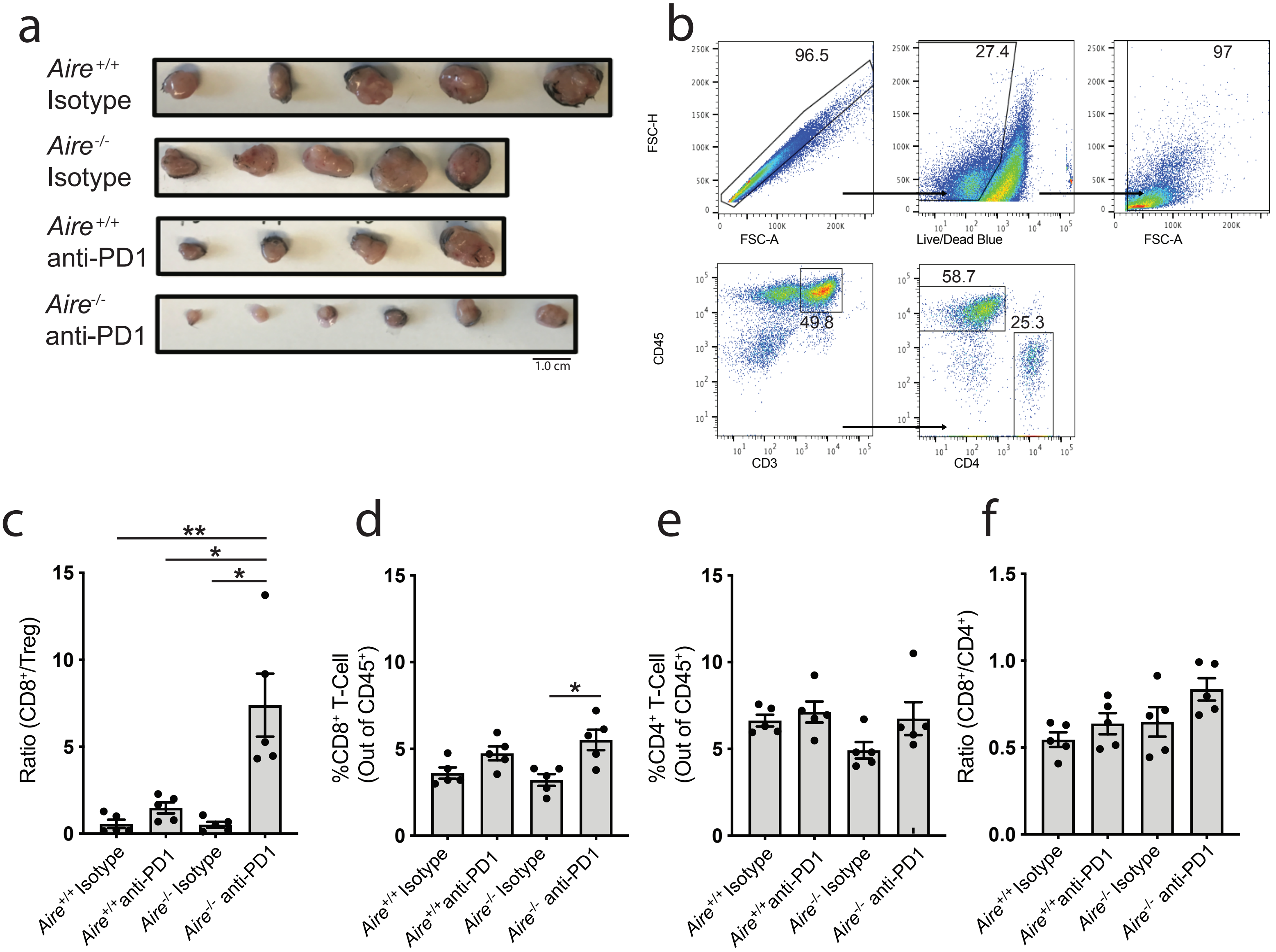

Supplementary Figure 1. Enhanced tumor killing in *Aire*$^{-/-}$ mice. Schematic depicting pictures of MC38 tumors from *Aire*$^{+/+}$ and *Aire*$^{-/-}$ treated with Isotype or anti-PD1. Scale bar represents 1.0 cm. **b** Gating strategy used for identifying tumor infiltrating lymphocytes. Cells were first gated on singlets, followed by gating on live cells, and forward/side scatter. **c** Ratio of intra-tumoral CD8^{+} T cells to T-regulatory cells (FOXP3^{+}) (*n=5* per group). **d-f** Percentage of splenic CD4^{+} or CD8^{+} T cells from *Aire*$^{+/+}$ and *Aire*$^{-/-}$ treated with isotype or anti-PD1 (*n=5* per group). Percentages were determined out of total CD45^{+} cells. Related to Figure 1C. Data are represented as mean ± SEM, (*, $P < 0.05$; **, $P < 0.01$; *** , $P < 0.001$), by one-way ANOVA with Tukey's test.

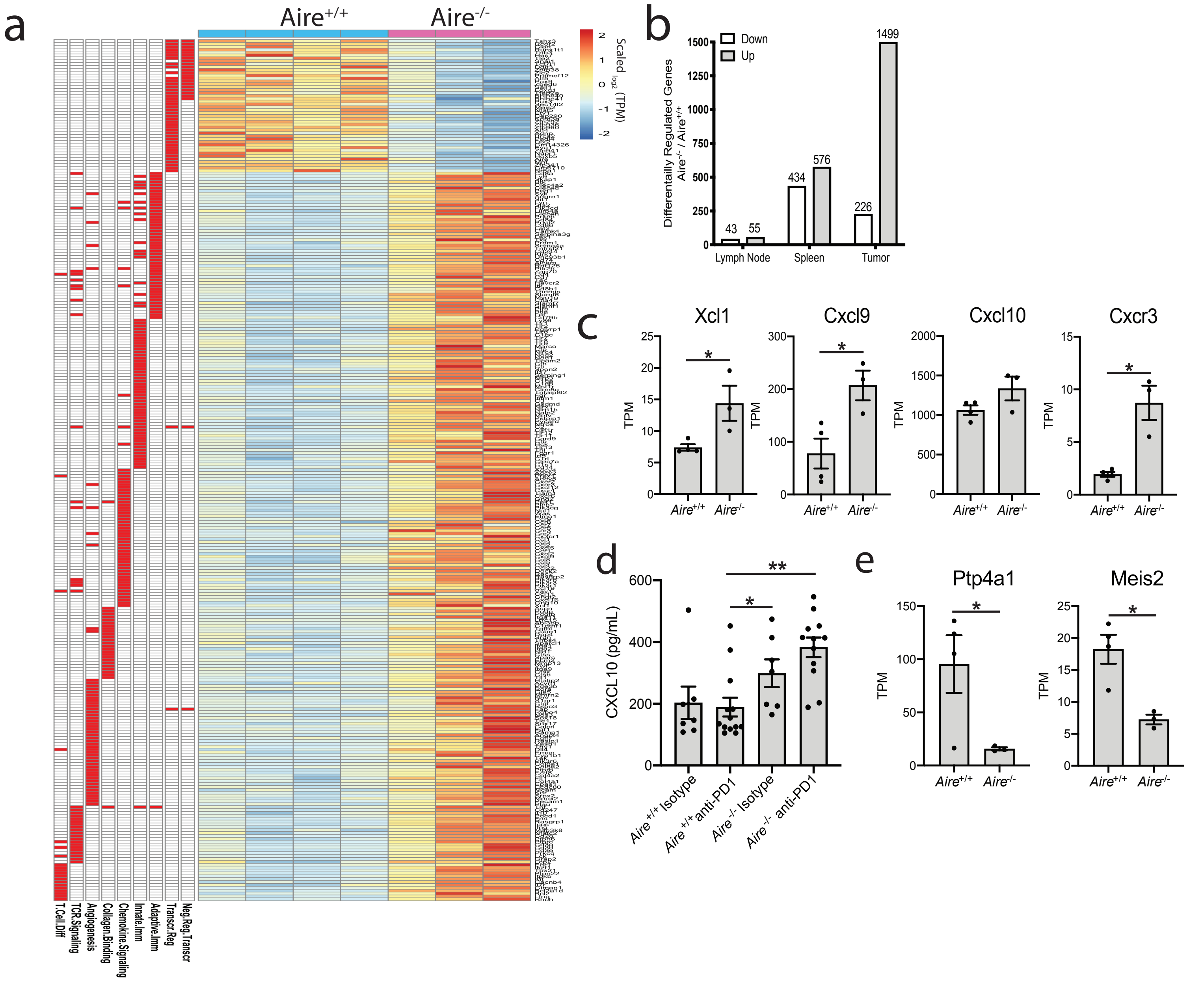

Supplementary Figure 2. Increased levels of differentially regulated genes in tumors from *Aire*$^{-/-}$ treated with anti-PD1. **a** Heatmap depicting the differentially regulated genes in tumors from *Aire*$^{+/+}$ (n=4) and *Aire*$^{-/-}$ (n=3) mice treated with anti-PD1. Differentially regulate genes were defined with log2 fold changes of 1.5 in either up or down direction and with p-values less than 0.01; TPM, transcripts per million. The expression value of each gene was divided by the median expression of the same gene across all samples. The genes found to be differentially regulated were grouped according to different biological pathways (columns on left shaded in red). **b** Number of differentially regulated genes in lymph nodes, spleen, and tumors from *Aire*$^{+/+}$ (n=4) and *Aire*$^{-/-}$ (n=3) mice. White and gray bars depict downregulated or upregualted genes, respectively. **c** Levels of Xcl1, Cxcl9, Cxcl10, and Cxcr3 in tumors from *Aire*$^{+/+}$ and *Aire*$^{-/-}$ mice. TPM, transcripts per million. **d** Levels of Cxcl10 in serum from *Aire*$^{+/+}$ and *Aire*$^{-/-}$ treated with isotype or anti-PD1 (n=7 for *Aire*$^{+/+}$ and *Aire*$^{-/-}$ treated with isotype, and n=12 for *Aire*$^{+/+}$ and *Aire*$^{-/-}$ treated with anti-PD1). **e** Levels of Ptp4a1, and Meis2 in tumors from *Aire*$^{+/+}$ and *Aire*$^{-/-}$ mice. Data are represented as mean ± SEM, (*, $P < 0.05$; **, $P < 0.01$; *** , $P < 0.001$), **a-c** and e by Student's t-test, and **d** by one-way ANOVA with Tukey's test.

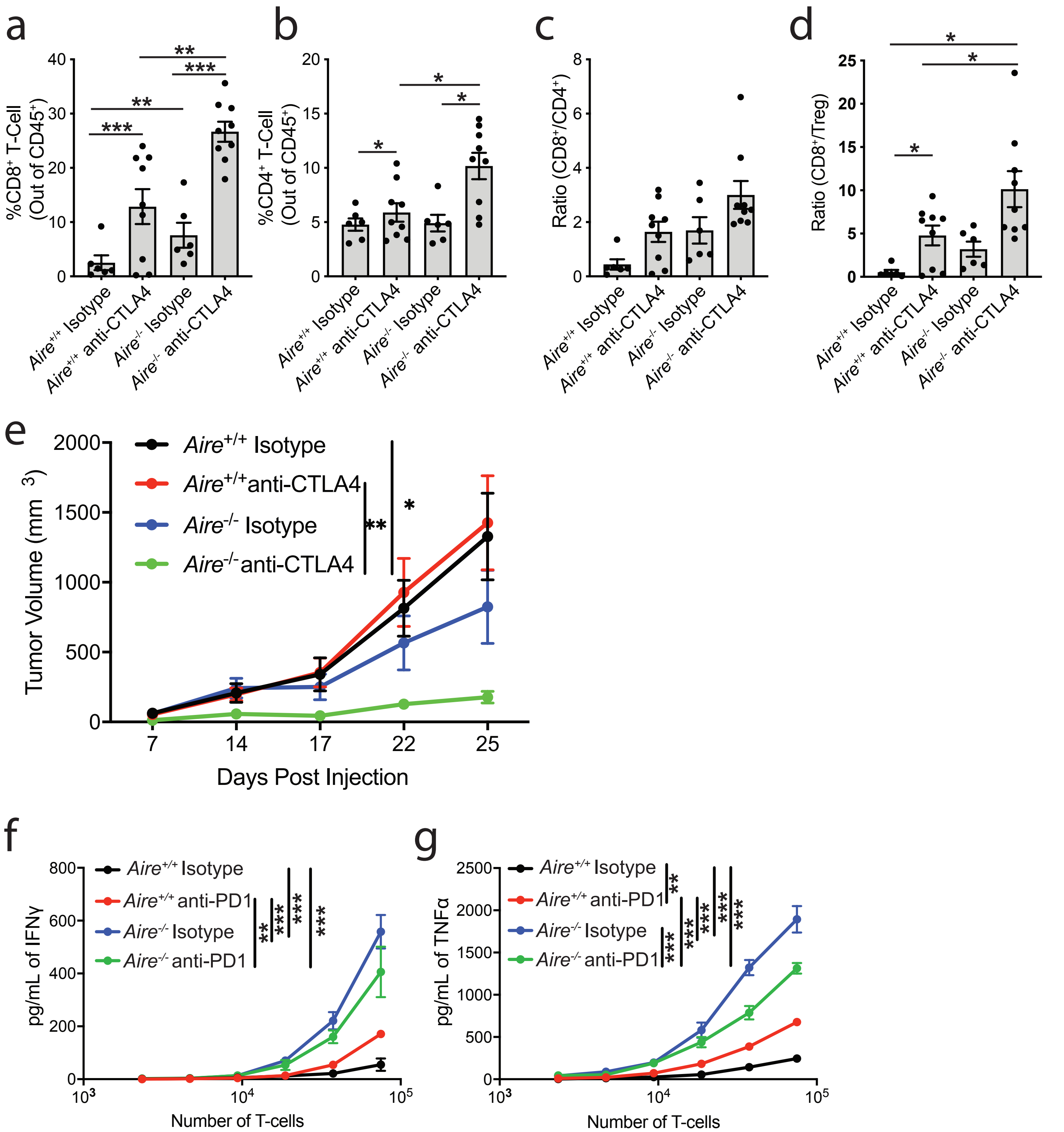

Supplementary Figure 3. Increased levels of $CD4^+$ and $CD8^+$ T-cells in tumors from Aire$^{-/-}$ mice treated with anti-CTLA4. a-b Percentage of intra-tumoral $CD8^+$ or $CD4^+$ T cells from *Aire*$^{+/+}$ and *Aire*$^{-/-}$ treated with isotype (n=6) or anti-CTLA4 (n=9). Percentages were determined out of total $CD45^+$ cells. **c** Ratio of intra-tumoral $CD8^+$ to $CD4^+$ T cells. **d** Ratio of intra-tumoral $CD8^+$ T cells to T-regulatory cells ($FOXP3^+$). **e** Growth kinetics of MC38 tumors in *Aire*$^{+/+}$ and *Aire*$^{-/-}$ treated with Isotype or anti-CTLA4 (n=7 per group). **f-g** Levels of IFNγ and TNFα in supernatants derived from the cytoxicity assay (n=3 per group). Related to Fig. 3c. Data are represented as mean ± SEM, (*, $P < 0.05$; **, $P < 0.01$; *** , $P < 0.001$), by one-way ANOVA with Tukey's test.

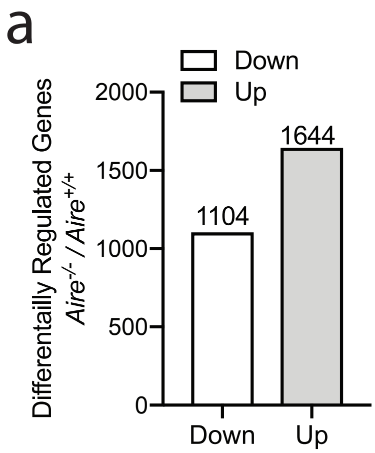

Supplementary Figure 4. Differentially upregulated genes tumors from *Aire*$^{-/-}$ treated with anti-CTLA4.
a Number of differentially regulated genes in tumors from *Aire*$^{+/+}$ and *Aire*$^{-/-}$ mice treated with anti-CTLA4. White and gray bars depict downregulated or upregualted genes, respectively. Related to Figure 4A.

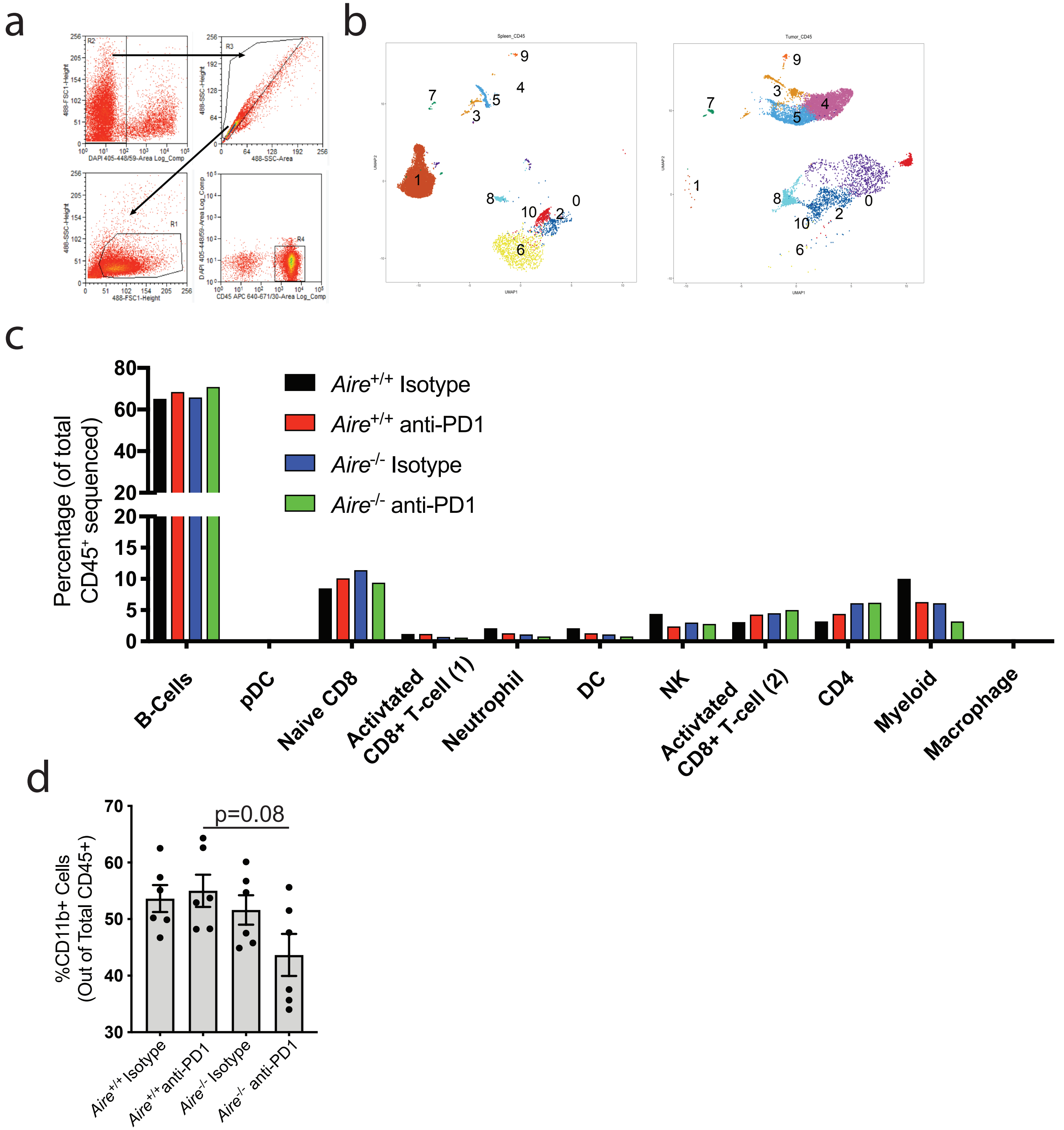

Supplementary Figure 5. scRNAseq of CD45+ cells in tumors from *Aire*$^{+/+}$ and *Aire*$^{-/-}$ mice. a Gating strategy for sorting CD45^{+} cells from spleens and tumors. Cells were first gated on live-cells by excluding DAPI-postive cells, followed by gating on singlets and forward/side scatter. CD45^{+} cells were sorted and collected for single-cell RNAseq. **b** UMAP plot depicting the unsupervised clustering of splenic CD45^{+} cells only. **c** Bar graph depicting the composition of each cluster identified in splenic CD45^{+} cells from *Aire*$^{+/+}$ and *Aire*$^{-/-}$ mice treated with Isotype or anti-PD1 (*n=6* per group). Data are represented as mean ± SEM, (*, P < 0.05; **, P < 0.01; *** , P < 0.001), by one-way ANOVA with Tukey's test.

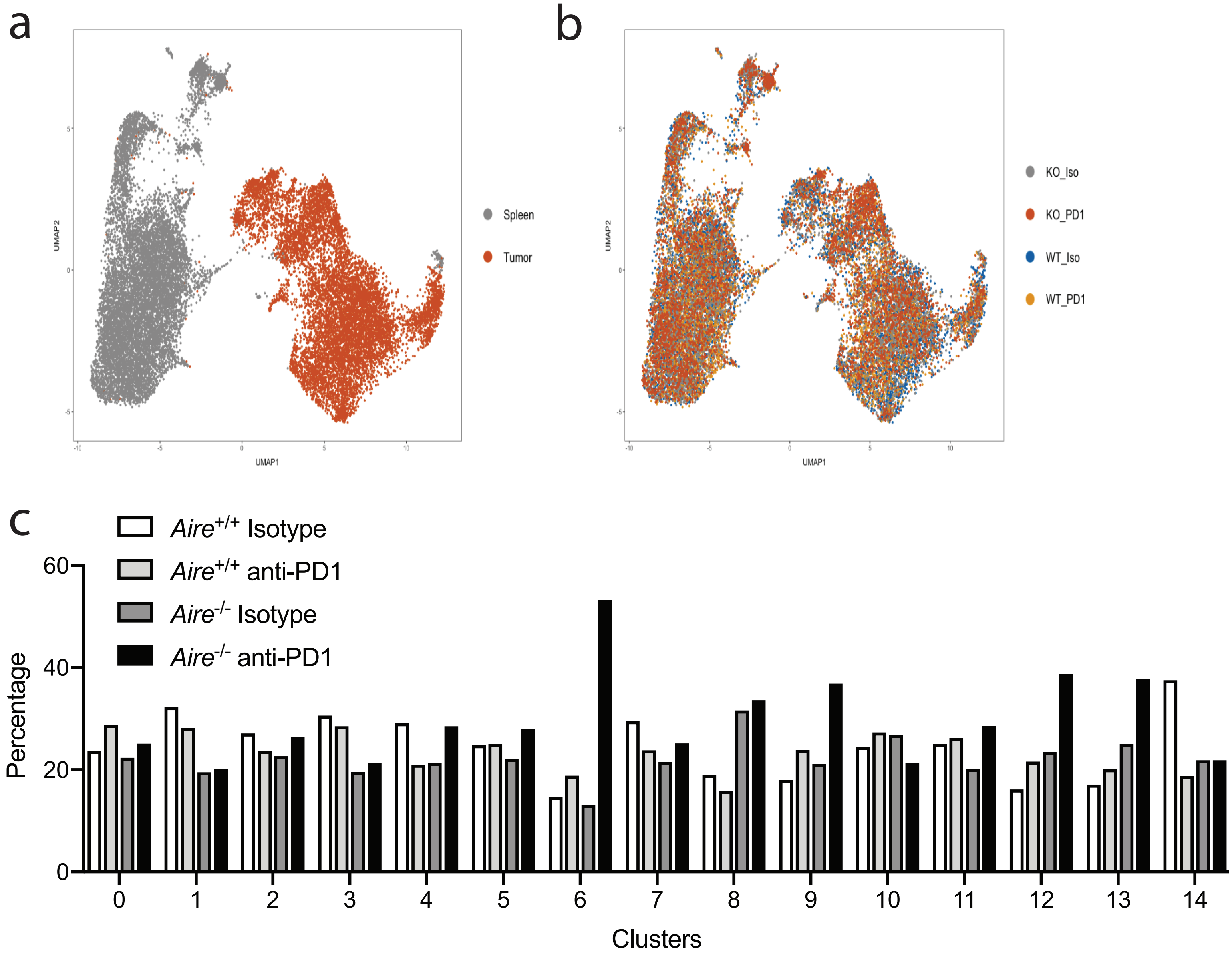

Supplementary Figure 6. Sub-clustering of CD8+ TILs from *Aire*$^{+/+}$ and *Aire*$^{-/-}$ mice. a UMAP plot depicting CD8^{+}T cells from spleens (gray) and tumors (orange). **b** UMAP plot depicting CD8^{+}T cells from all conditions tested represented in different colors. **c** Percentage of each CD8^{+} T cells in *Aire*$^{+/+}$ and *Aire*$^{-/-}$ treated with with Isotype or anti-PD1 in each of the clusters identified. Related to Figure 6C. **e** Expression values (fold change) of genes upregulated or downregulated in tumors from *Aire*$^{+/+}$ and *Aire*$^{-/-}$ treated with with anti-PD1 in cluster 6. **f** Expression values (fold change) of genes upregulated or down-regulated in tumors from *Aire*$^{+/+}$ and *Aire*$^{-/-}$ treated with with anti-PD1 in cluster 9.

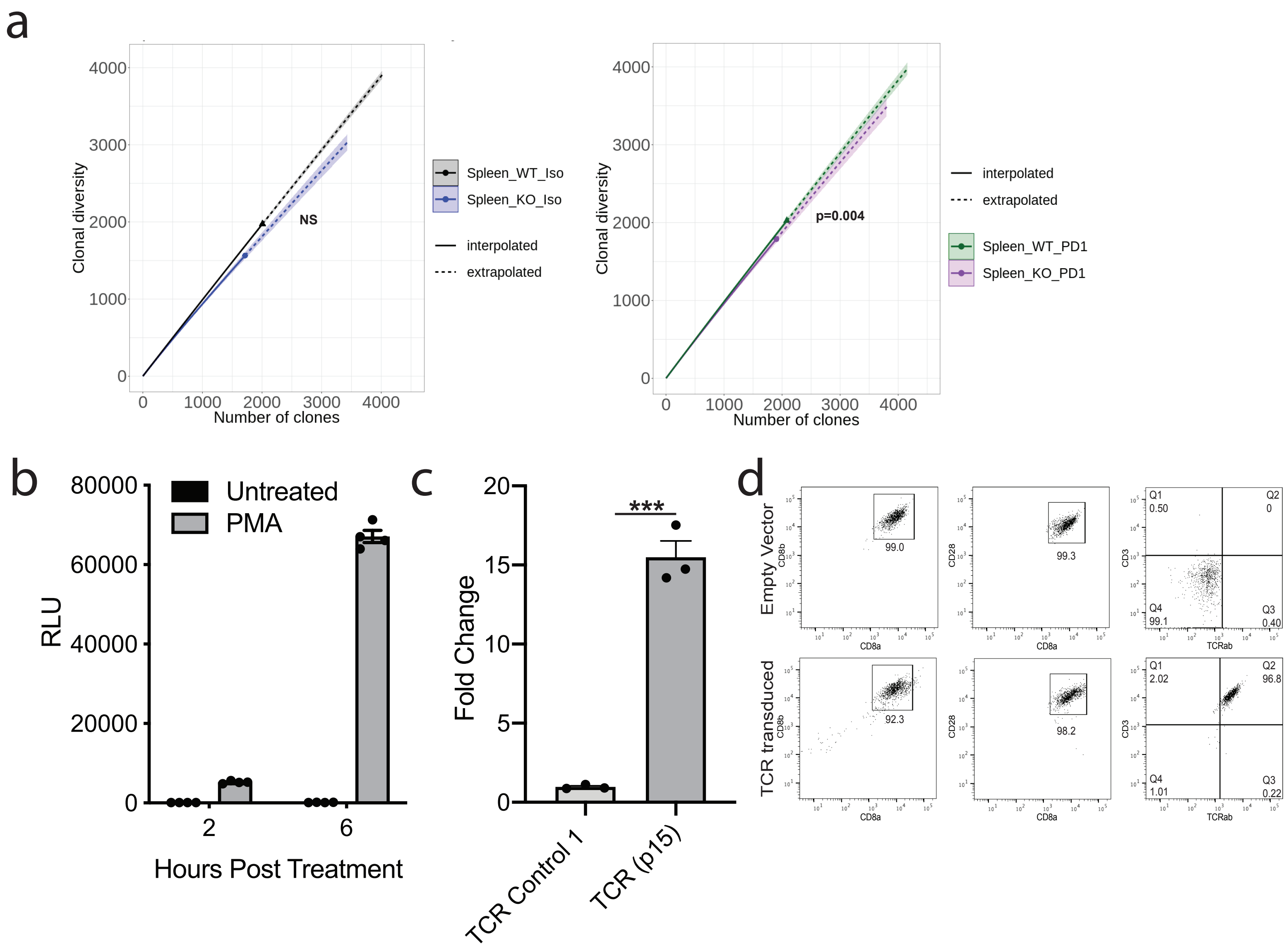

Supplementary Figure 7. Clonal and diversity analysis of CD8$^+$ TILs. a Shannon diversity was calculated for clones defined by the CDR3 sequences of TCR alpha and beta chains in spleens from *Aire*$^{+/+}$ and *Aire*$^{-/-}$ MC38 tumor-bearing mice treated with isotype or anti-PD1 antibodies on Day 19. Data were bootstrapped 50 times at the indicated sample sizes along the x-axis. Interpolated lines (solid) were determined based on sample sizes smaller than the actual sample size. Extrapolated lines (dashed) were determined with sample sizes larger than the actual sample size. Extrapolated lines (dashed) were determined with sample sizes larger than the actual sample size. **b** Activation of JRT3 cells with PMA leads to production of Luciferase after 2 and 6 hours (*n=4* per group). **c** Fold change in activation of the engineered T cells expressing the a TCR recognizing p15. Engineered T-cells were mixed with tumor cells and luciferase activity was assessed 6 hours later (*n=3* per group). Fold change in RLU activity was determined over the parental cell line. **d** Representative FACS plots of JRT3 cells expressing the cloned TCR. Cells were transduced with lentiviruses expressing either a TCR or none as a control. Cells were stained for CD8α, CD8ß, CD28, CD3, and TCRα/ß and expression was confirmed using flow-cytometry. Data are represented as mean ± SEM, (*, $P < 0.05$; **, $P < 0.01$; *** , $P < 0.001$), by one-way ANOVA with Tukey's test.

Supplementary Tables

Supplementary Table 1. Number of CD45^{+} cells from tumors or spleens from *Aire*$^{+/+}$ and *Aire*$^{-/-}$ mice.

	Tissue	Sorted	Treatment	Raw Cells	QC Pass
Aire$^{+/+}$	Tumor	CD45	Isotype	2936	2930
		CD45	anti-PD1	2211	2208
	Spleen	CD45	Isotype	2119	2095
		CD45	anti-PD1	2117	2088
Aire$^{-/-}$	Tumor	CD45	Isotype	1860	1855
		CD45	anti-PD1	2076	2075
	Spleen	CD45	Isotype	2048	2023
		CD45	anti-PD1	2402	2393

Raw cell number represents the number of cells sequenced, while QC pass represents the number of cells that passed quality control criteria outlined in the Methods section.

Supplementary Table 2. Upregulated and downregulated genes in activated CD8$^+$ TILs from *Aire*$^{-/-}$ mice treated with anti-PD1 (Cluster 0).

Gene	Fold Change	Description	Cluster Expression (%)	Expression Out of Cluster (%)
Gzmb	1.89	granzyme B	0.853	0.708
Gem	1.85	GTP binding protein (gene overexpressed in skeletal muscle)	0.818	0.611
BC002163	1.85	NADH dehydrogenase Fe-S protein 5 pseudogene	0.614	0.104
Prf1	1.84	perforin 1 (pore forming protein)	0.803	0.639
Ccl5	1.81	chemokine (C-C motif) ligand 5	0.705	0.59
Serpine2	1.78	serine (or cysteine) peptidase inhibitor, clade E, member 2	0.314	0.16
Odc1	1.59	ornithine decarboxylase, structural 1	0.907	0.722
Cap1	1.5	CAP, adenylate cyclase-associated protein 1 (yeast)	0.966	0.91
Fcgr2b	-1.5	Fc receptor, IgG, low affinity IIb	0.025	0.097
Alox5ap	-1.52	arachidonate 5-lipoxygenase activating protein	0.017	0.09
Zfp36	-1.53	zinc finger protein 36	0.55	0.639
Gpr183	-1.53	G protein-coupled receptor 183	0.56	0.75
Lgmn	-1.54	legumain	0.037	0.111
Rgs1	-1.59	regulator of G-protein signaling 1	0.826	0.819
Mafb	-1.65	v-maf musculoaponeurotic fibrosarcoma oncogene family, protein B (avian)	0.027	0.125
Tsc22d3	-1.68	TSC22 domain family, member 3	0.636	0.806
Rnaset2b	-1.72	ribonuclease T2B	0.197	0.674
Klf2	-1.74	Kruppel-like factor 2 (lung)	0.285	0.424
Lyz2	-1.84	lysozyme 2	0.057	0.132
Il1b	-1.94	interleukin 1 beta	0.015	0.111
Apoe	-2.36	apolipoprotein E	0.052	0.16
Tgfbi	-2.41	transforming growth factor, beta induced	0.049	0.139
Tyrobp	-2.68	TYRO protein tyrosine kinase binding protein	0.066	0.174
Slfn4	-2.81	schlafen 4	0.002	0.076
Cd74	-4.4	CD74 antigen (invariant polypeptide of major histocompatibility complex, class II antigen-associated)	0.361	0.465

Fold change in gene expression over the TILs from *Aire*$^{+/+}$ mice treated with anti-PD1. Cluster expression refers to the percentage of cells within the cluster expressing each gene, while expression out of cluster refers to the percentage of cells in all other clusters expressing each gene. FDR cut-off of 0.05.

Supplementary Table 3. Upregulated and downregulated genes in activated $CD8^+$ TILs from *Aire*$^{-/-}$ mice treated with anti-PD1 (Cluster 2).

Gene	Fold Change	Description	Cluster Expression (%)	Expression Out of Cluster (%)
Lmna	2.35	lamin A	0.632	0.416
Cd74	2.12	CD74 antigen (invariant polypeptide of major histocompatibility complex, class II antigen-associated)	0.297	0.192
Fos	1.87	FBJ osteosarcoma oncogene	0.696	0.584
Gzma	1.72	granzyme A	0.378	0.256
Cd8b1	1.72	CD8 antigen, beta chain 1	0.708	0.504
Itgae	1.7	integrin alpha E, epithelial-associated	0.359	0.184
Samhd1	1.68	SAM domain and HD domain, 1	0.822	0.696
Egr1	1.66	early growth response 1	0.442	0.32
Csf1	1.64	colony stimulating factor 1 (macrophage)	0.292	0.128
Ctla4	1.6	cytotoxic T-lymphocyte-associated protein 4	0.572	0.416
Cd8a	1.59	CD8 antigen, alpha chain	0.689	0.52
Cxcr6	1.58	chemokine (C-X-C motif) receptor 6	0.698	0.56
S100a4	1.58	S100 calcium binding protein A4	0.694	0.504
Stat1	1.53	signal transducer and activator of transcription 1	0.836	0.816
Cap1	1.5	CAP, adenylate cyclase-associated protein 1 (yeast)	0.905	0.752
Bcl2	-1.52	B cell leukemia/lymphoma 2	0.542	0.736
Klra7	-1.57	killer cell lectin-like receptor, subfamily A, member 7	0.052	0.144
Tsc22d3	-1.67	TSC22 domain family, member 3	0.684	0.904
TrnC	-1.72	tRNA cysteine, mitochondrial	0.418	0.688
Rnaset2b	-1.97	ribonuclease T2B	0.202	0.688
Klf2	-1.97	Kruppel-like factor 2 (lung)	0.71	0.848
Tnfrsf4	-2.28	tumor necrosis factor receptor superfamily, member 4	0.093	0.176
Ecm1	-2.86	extracellular matrix protein 1	0.052	0.12
Hspa1a	-3.17	heat shock protein 1A	0.024	0.072

Fold change in gene expression over the TILs from *Aire*$^{+/+}$ mice treated with anti-PD1. Cluster expression refers to the percentage of cells within the cluster expressing each gene, while expression out of cluster refers to the percentage of cells in all other clusters expressing each gene. FDR cut-off of 0.05.

Supplementary Table 4. Upregulated and downregulated genes in intratumoral NK cells from *Aire*$^{-/-}$ mice treated with anti-PD1 (Cluster 8).

Gene	Fold Change	Description	Cluster Expression (%)	Expression Out of Cluster (%)
Egr1	1.87	early growth response 1	0.515	0.35
Plac8	1.63	placenta-specific 8	0.599	0.464
Pim1	1.55	proviral integration site 1	0.819	0.7
BC002163	1.51	NADH dehydrogenase Fe-S protein 5 pseudogene	0.478	0.129
Rnaset2b	-1.62	ribonuclease T2B	0.344	0.793
Klf2	-1.84	Kruppel-like factor 2 (lung)	0.625	0.729

Fold change in gene expression over the NK cells from *Aire*$^{+/+}$ mice treated with anti-PD1. Cluster expression refers to the percentage of cells within the cluster expressing each gene, while expression out of cluster refers to the percentage of cells in all other clusters expressing each gene. FDR cut-off of 0.05.

Supplementary Table 5. Number of CD8$^+$ T-cells sequenced from tumors or spleens from *Aire*$^{+/+}$ and *Aire*$^{-/-}$ mice.

	Tissue	Sorted	Treatment	Raw Cells	QC Pass	Cells with TCRA and TCRB sequences
Aire$^{+/+}$	Tumor	CD8	Isotype	2688	2684	1596
		CD8	anti-PD1	2222	2221	1637
	Spleen	CD8	Isotype	2902	2898	2007
		CD8	anti-PD1	2966	2944	2081
Aire$^{-/-}$	Tumor	CD8	Isotype	1860	1858	1345
		CD8	anti-PD1	2214	2212	1968
	Spleen	CD8	Isotype	2355	2339	1715
		CD8	anti-PD1	2633	2629	1901

Raw cell number represents the number of cells sequenced, while QC pass represents the number of cells that passed quality control criteria outlined in the Methods section. Last column lists the number of cells with complete TCR alpha and beta sequences.

Supplementary Table 6. Sequences of TCRs isolated from tumors CD8+ TILs in *Aire*$^{-/-}$ mice treated with anti-PD1.

Name	Sequence	Size	Percentage
TCR 1.1, TCR 1.2	TRA:CALGETASLGKLQF;TRA:CAMREGSTGGNNKLTF;TRB:CASSSRGLYEQYF	138	9.67
TCR2	TRA:CATDGGNYAQGLTF;TRB:CASSFRNANSDYTF	100	7.00
TCR3	TRA:CAVSGDTNAYKVIF;TRB:CASSPDWGASSQNTLYF	47	3.29
TCR4	TRA:CALGDSDTNAYKVIF;TRB:CASSYRQGNYAEQFF	40	2.80
		Total	22.76

Size represents the number of clones identified that share the same TCR alpha and beta sequences. Percentage was determined by dividing the number of clones by the total number of $CD8^+$ TILs.
