# Supplementary material for: Absence of central tolerance in Aire-deficient mice synergizes with immune-checkpoint inhibition to enhance antitumor responses
Source: Commun Biol. 2020 Jul 8;3:355. doi: 10.1038/s42003-020-1083-1 (PMC7343867; doi:10.1038/s42003-020-1083-1)
Supplement: Supplementary file 2 — Description of Additional Supplementary Files [file 42003_2020_1083_MOESM2_ESM.pdf]

Description of Additional Supplementary Files

File Name: Supplementary Data 1
Description: Bulk RNAseq data in MC38 tumors from *Aire*$^{+/+}$ and *Aire*$^{-/-}$ treated with Isotype or anti-PD1 antibodies. Data are shown as TPM (transcripts per million). Related to Figure 2.

File Name: Supplementary Data 2
Description: Bulk RNAseq data in B16.F10 tumors from *Aire*$^{+/+}$ and *Aire*$^{-/-}$ treated with Isotype or anti-CTLA4 antibodies. Data are shown as TPM (transcripts per million). Related to Figure 4.

File Name: Supplementary Data 3
Description: Upregulated genes in tumor macrophages from *Aire*$^{-/-}$ mice treated with anti-PD1. Fold change in gene expression over the macrophages cells from *Aire*$^{+/+}$ mice treated with anti-PD1. Cluster expression refers to the percentage of cells within the cluster expressing each gene, while expression out of cluster refers to the percentage of cells in all other clusters expressing each gene. FDR cut-off of 0.05.

File Name: Supplementary Data 4
Description: Upregulated genes in CD8^{+} TILs from *Aire*$^{-/-}$ mice treated with anti-PD1 (Cluster 6). Fold change in gene expression in CD8^{+} TILs from *Aire*$^{-/-}$ mice treated with anti-PD1 over the other conditions tested in Cluster 6. Cluster expression refers to the percentage of cells within the cluster expressing each gene, while expression out of cluster refers to the percentage of cells in all other clusters expressing each gene. FDR cut-off of 0.05.

File Name: Supplementary Data 5
Description: Description: Upregulated genes in CD8^{+} TILs from *Aire*$^{-/-}$ mice treated with anti-PD1 (Cluster 9). Fold change in gene expression in CD8^{+} TILs from *Aire*$^{-/-}$ mice treated with anti-PD1 over the other conditions tested in Cluster 9. Cluster expression refers to the percentage of cells within the cluster expressing each gene, while expression out of cluster refers to the percentage of cells in all other clusters expressing each gene. FDR cut-off of 0.05.

File Name: Supplementary Data 6
Description: scRNAseq results from CD45^{+} cells sequenced from MC38 tumors from *Aire*$^{+/+}$ and *Aire*$^{-/-}$ treated with Isotype or anti-PD1 antibodies. Data are shown as fold-change over all conditions and are separated by tabs. Related to Figure 5.

File Name: Supplementary Data 7
Description: scRNAseq results from CD8^{+} TILs cells sequenced from MC38 tumors from *Aire*$^{+/+}$ and *Aire*$^{-/-}$ treated with Isotype or anti-PD1 antibodies. Data are shown as fold-change over all conditions and are separated by tabs. Related to Figure 6.

File Name: Supplementary Data 8
Description: Sequences of TCR alpha and beta of the $CD8^+$ TILs identified.

File Name: Supplementary 9
Description: Source data of all the graphs.
