# Supplementary material for: Absence of central tolerance in Aire-deficient mice synergizes with immune-checkpoint inhibition to enhance antitumor responses
Source: Commun Biol. 2020 Jul 8;3:355. doi: 10.1038/s42003-020-1083-1 (PMC7343867; doi:10.1038/s42003-020-1083-1)
Supplement: Supplementary file 12 — Reporting Summary [file 42003_2020_1083_MOESM12_ESM.pdf]

nature research

Corresponding author(s): SOKOL HAXHINASTO

Last updated by author(s): Jun 8, 2020

Reporting Summary

Nature Research wishes to improve the reproducibility of the work that we publish. This form provides structure for consistency and transparency in reporting. For further information on Nature Research policies, see Authors & Referees and the Editorial Policy Checklist.

Statistics

For all statistical analyses, confirm that the following items are present in the figure legend, table legend, main text, or Methods section.

n/a	Confirmed	
☐	☒	The exact sample size (*n*) for each experimental group/condition, given as a discrete number and unit of measurement
☐	☒	A statement on whether measurements were taken from distinct samples or whether the same sample was measured repeatedly
☐	☒	The statistical test(s) used AND whether they are one- or two-sided<br>*Only common tests should be described solely by name; describe more complex techniques in the Methods section.*
☐	☒	A description of all covariates tested
☒	☐	A description of any assumptions or corrections, such as tests of normality and adjustment for multiple comparisons
☐	☒	A full description of the statistical parameters including central tendency (e.g. means) or other basic estimates (e.g. regression coefficient) AND variation (e.g. standard deviation) or associated estimates of uncertainty (e.g. confidence intervals)
☐	☒	For null hypothesis testing, the test statistic (e.g. *F*, *t*, *r*) with confidence intervals, effect sizes, degrees of freedom and *P* value noted<br>*Give P values as exact values whenever suitable.*
☒	☐	For Bayesian analysis, information on the choice of priors and Markov chain Monte Carlo settings
☒	☐	For hierarchical and complex designs, identification of the appropriate level for tests and full reporting of outcomes
☒	☐	Estimates of effect sizes (e.g. Cohen's *d*, Pearson's *r*), indicating how they were calculated

Our web collection on statistics for biologists contains articles on many of the points above.

Software and code

Policy information about availability of computer code

Data collection	For Bulk RNAseq: RNAseq read mapping and statistical analysis of differentially expressed RNA. Raw sequence data (BCL files) were converted to FASTQ format via Illumina bcl2fastq v2.17. Reads were decoded based on their barcodes and read quality was evaluated with FastQC (http://www.bioinformatics.babraham.ac.uk/projects/fastqc/). Reads were mapped to the mouse genome (mm10) using ArrayStudio® software (OmicSoft®, Cary, NC) allowing two mismatches. Reads mapped to the exons of a gene were summed at the gene level. Differential expressed genes were identified by the DESeq2 R package (Love et al., 2014) and significantly perturbed genes were defined with fold changes of at least 1.5 in either up or down direction and with p-values less than 0.01.<br>For scRNAseq: 387 Single-cell RNA sequencing, and read mapping. Single cell suspension of tumors were sorted for CD45+ or CD8+ 388 and collected into tubes containing PBS with 0.04% BSA. The cell suspensions were loaded on a Chromium Single Cell Instrument (10X Genomics) and RNA libraries were prepared using Chromium Single Cell 3' Library, Gel Beads & Multiplex Kit (10X Genomics). Paired-end sequencing was performed on Illumina NextSeq500 where Read 1 was used for unique molecular identifier (UMI) and cell barcode while Read 2 was used for 55-bp transcript read. Sample demultiplexing, alignment, filtering, and UMI counting were performed on Cell Ranger Single-Cell Software Suite (10X Genomics).
Data analysis	Single-cell data analysis. Single-cell analysis was carried out using version 2 of the Seurat R package (Butler et al., 2018, R Core Team, 2018). Cells with fewer than 200 genes detected or over 20% of reads mapping to mitochondrial genes were discarded from analysis. Gene expression values for each cell were normalized and scaled and variation due to cell cycle stage and mitochondrial rate were regressed out as described previously (Butler et al., 2018). The number of UMI was also regressed out to correct for variation in sampling depth of these cells. The genes used for principal component analysis (PCA) were the 1000 genes with the highest dispersion (variance to mean ratio) for genes with mean UMI between 0.0125 and 8 and variance above 0.5. Genes were divided into 20 bins of equal width based on their average expression and dispersion 14 z-scores were calculated within these bins. Cells were then partitioned into clusters (Seurat FindClusters function) and visualized using the t-distributed stochastic neighbor embedding (t406 SNE) algorithm (Seurat RunTSNE function) as described previously (Butler et al., 2018). The first 15 principal components were used to run the t-SNE dimensionality reduction. The FindClusters function was run with a resolution parameter of 0.4, resulting in 14 clusters of cells. These clusters corresponded to naïve CD8+ T cells, B cells, activated CD8+ T cells, macrophages, CD8+ effector T cells, myeloid cells, dendritic cells, natural killer cells, CD4+ regulatory T cells, plasmacytoid dendritic cells, and neutrophils. Cluster cell type identities were

determined by examining marker genes specifically expressed more highly in each cluster (Seurat FindAllMarkers function) and expression of known immune marker genes (Seurat FeaturePlot function).

For manuscripts utilizing custom algorithms or software that are central to the research but not yet described in published literature, software must be made available to editors/reviewers. We strongly encourage code deposition in a community repository (e.g. GitHub). See the Nature Research guidelines for submitting code & software for further information.

Data

Policy information about availability of data

All manuscripts must include a data availability statement. This statement should provide the following information, where applicable:

- Accession codes, unique identifiers, or web links for publicly available datasets
- A list of figures that have associated raw data
- A description of any restrictions on data availability

The datasets generated during and/or analysed during the current study are not publicly available due to [these datasets including GOI accession codes, etc. will be made available prior to publication] but are available from the corresponding author on reasonable request.

Field-specific reporting

Please select the one below that is the best fit for your research. If you are not sure, read the appropriate sections before making your selection.

☒ Life sciences ☐ Behavioural & social sciences ☐ Ecological, evolutionary & environmental sciences

For a reference copy of the document with all sections, see nature.com/documents/nr-reporting-summary-flat.pdf

Life sciences study design

All studies must disclose on these points even when the disclosure is negative.

Sample size	For experiments involving tumors, sample sizes ranged from 8-10 due to the variable nature of tumors.
Data exclusions	No data were excluded
Replication	All experiments were reproduced a minimum of three times with similar results.
Randomization	Allocation of animals into each experimental group was random.
Blinding	All animals were randomly assigned into experimental groups. Tumor volume measurements were obtained blindly.

Reporting for specific materials, systems and methods

We require information from authors about some types of materials, experimental systems and methods used in many studies. Here, indicate whether each material, system or method listed is relevant to your study. If you are not sure if a list item applies to your research, read the appropriate section before selecting a response.

Materials & experimental systems

n/a	Involved in the study	
☐	☒	Antibodies
☐	☒	Eukaryotic cell lines
☒	☐	Palaeontology
☐	☒	Animals and other organisms
☒	☐	Human research participants
☒	☐	Clinical data

Methods

n/a	Involved in the study	
☒	☐	ChIP-seq
☐	☒	Flow cytometry
☒	☐	MRI-based neuroimaging

Antibodies

Antibodies used	FoxP3-e450 (Clone FJK-16s, eBioscience, Cat. #48-5773-82), CD45-BV510 (Clone 30-F11, Biolegend, Cat. #103138), PD1-BV605 (Clone JA3, BD Bioscience, Cat. #563059), NK1.1-BV650 (Clone PKI36, BD Bioscience, Cat. #564143), GITR362 BV711 (Clone DTA-1, BD Bioscience, Cat. #563390), CD4-BV786 (Clone RM4-5, BD Bioscience, Cat. #563727), CD11B-BUV395 (Clone M1/70, BD Bioscience, Cat. #563553), CD44-BUV737 (Clone IM7, BD Bioscience, Cat. #564392), CD8-BUV805 (Clone 53-6.7, BD Bioscience, Cat. #564920), CD3-Alexa 700 (Clone 17A2, eBioscience, Cat. #56-0032-82), Lag3-APC-Cy7 (Clone eBioC9B7W, eBioscience, Cat. #47-2231-82), Tim3-PE (Clone RMT3-23 eBioscience, Cat. #12-5870-82), CTLA4-PE-CF594 (Clone UC10-4F10-11, BD Bioscience, Cat.#564332), 2B4-PE-Cy7 (Clone m2B4(B6)458.1, Biolegend, Cat. #133512), Live/Dead Blue369 Fixable Blue Dye (Invivogen, Cat. #L-23105), KLRG1-FITC (Clone 2F1, eBioscience, Cat. #11-370 5893-82).

Validation	All antibodies have been validated by the Regeneron Pharmaceuticals Flow Cytometry Core. In addition, the manufacturer's (Biolegend, eBioscience, and BD Bioscience) have extensively validated the antibodies used.

Eukaryotic cell lines

Policy information about cell lines

Cell line source(s)	The cell lines were obtained from the Regeneron Pharmaceuticals Tissue Culture Core which obtains lines from ATCC and other sources.
Authentication	The cell lines used were authenticated by the Regeneron Pharmaceuticals Tissue Culture Core.
Mycoplasma contamination	All cell lines used tested negative for Mycoplasma contamination.
Commonly misidentified lines (See ICLAC register)	No misidentified cell lines were used

Animals and other organisms

Policy information about studies involving animals; ARRIVE guidelines recommended for reporting animal research

Laboratory animals	Wild-type or Aire knockout mice (Source: Jackson Laboratories, Stock # 004743). Sex: Female, Age: 8 weeks old
Wild animals	No wild animals were used in this study
Field-collected samples	No field-collected samples were used in this study
Ethics oversight	All experiments were approved by Regeneron Pharmaceuticals, Inc. IACUC.

Note that full information on the approval of the study protocol must also be provided in the manuscript.

Flow Cytometry

Plots

Confirm that:

☒ The axis labels state the marker and fluorochrome used (e.g. CD4-FITC).

☒ The axis scales are clearly visible. Include numbers along axes only for bottom left plot of group (a 'group' is an analysis of identical markers).

☒ All plots are contour plots with outliers or pseudocolor plots.

☒ A numerical value for number of cells or percentage (with statistics) is provided.

Methodology

Sample preparation	Tumors were enzymatically dissociated into single cell suspensions using Miltenyi Biotec's Mouse Tumor Dissociation Kit (Stock 130-096-730). Single cell suspensions of spleen and lymph nodes were prepared by mechanical dissociation.
Instrument	Fortessa X-20
Software	Samples were collected using FACSdiva and analyzed on Flowjo 10.4.
Cell population abundance	The relevant cell populations comprising of CD45+ infiltrates in tumors accounted for >20% of the cell population. From this population T cells accounted for ~40% of the population.
Gating strategy	Cells were first gated to remove all cell debris by doing SSA/FSA. The cells were then gated to remove any doublets, followed by a gate to remove any dead cells. The boundaries between live and dead cells was determined using the appropriate controls (e.g. unstained).

☒ Tick this box to confirm that a figure exemplifying the gating strategy is provided in the Supplementary Information.
